# Numerical investigation of flexible Purcell-like integrated microfluidic pumps

**DOI:** 10.1063/5.0109263

**Published:** 2022-10-25

**Authors:** Jacob L. Binsley, Stefano Pagliara, Feodor Y. Ogrin

**Affiliations:** 1 Department of Physics and Astronomy, University of Exeter, Exeter EX4 4QL, United Kingdom; 2 Living Systems Institute and Biosciences, University of Exeter, Exeter EX4 4QD, United Kingdom

## Abstract

Integrating miniature pumps within microfluidic devices is crucial for advancing point-of-care diagnostics. Understanding the emergence of flow from novel integrated pumping systems is the first step in their successful implementation. A Purcell-like elasto-magnetic integrated microfluidic pump has been simulated in COMSOL Multiphysics and its performance has been investigated and evaluated. An elastic, cilia-like element contains an embedded magnet, which allows for actuation via a weak, uniaxial, sinusoidally oscillating, external magnetic field. Pumping performance is correlated against a number of variables, such as the frequency of the driving field and the proximity of the pump to the channel walls, in order to understand the emergence of the pumping behavior. Crucially, these simulations capture many of the trends observed experimentally and shed light on the key interactions. The proximity of the channel walls in the in-plane direction strongly determines the direction of net fluid flow. This characterization has important implications for the design and optimization of this pump in practical applications.

## INTRODUCTION

In recent decades, there has been a surge in the development of integrated pumping solutions for lab-on-a-chip (LOC) microfluidic devices.^1,2^ This class of devices are integral to the progression of point-of-care (POC) testing^3,4^ and are abundant in fields such as biomedicine.^5–7^

Many simple diagnostic tests can be built to rely on passive flow generation such as capillary action^8,9^ including, famously, the lateral flow immunoassays used for rapid testing of COVID-19.^10^ This class of pumping solution can be inexpensively fabricated and readily forgo external connections or requirements of power delivery. However, a range of common flaws are yet to be satisfactorily tackled, such as difficulties in maintaining a constant flow rate or prolonged operation times.^11^ This is important for experiments involving culturing bacteria in deterministic environments, eliminating external stresses, and enabling long term cell trapping through constant pressure.^7,12,13^

Therefore, there is still much to be gained from the development of active pumping solutions.^1,2^ With access to ideal laboratory resources, flow may commonly be generated using an external, tabletop pumping system.^14,15^ Such external systems are, however, inappropriate for use in conjunction with POC devices due to their limited capacity for distribution owing to their size and expense.

Ideally, active fluid flow can be generated from miniaturized pumping systems integrated within the LOC device. However, it is non-trivial to create significant and controllable fluid flow in the low Reynolds number regime using miniature on-board components typical of microfluidic devices.^16^ This is particularly true under the constraint that such components are fabricated using the same processes as the chips themselves, relying heavily on planar monolithography.^17^

Therefore, this pursuit of generating net motion in the Stokes regime has produced a plethora of solutions. Potential solutions have been found making use of magnetic,^2,18–27^ electric,^28^ and inertial fields;^6^ chemical and optical means;^29–34^ mechanical displacements;^2,35^ and acoustic waves.^36–40^

Much of this research is complemented by the use of computational fluid dynamics (CFD). CFD is an integral part of microfluidic advancement in the 21st century, which is used to complement experimental works^41^ or as a stand alone investigation.^42^ This is particularly prominent with regard to micromixing^43,44^ and inertial microfluidics^45^ and is applied to support experimental work into microfluidic pumps.

Simulation results often allow further insight into experimental systems without the concern of fabrication tolerances. With the ability to look into the transient evolution of streamlines and phase relationships between pressure and flow, we can more accurately characterize complex systems such as Purcell-like magnetoelastic pumps. This allows for a more quantitative view of optimization criteria as well as an important verification of the experiments, such as has been used in previous cilia-like^41,46–48^ or pumping systems.^42^

Recently, we produced an experimental device that showed successful flow production as an integrated microfluidic pump.^49^ This consisted of an elasto-magnetic pump driven by an oscillating magnetic field and fabricated using the common manufacturing method of monolayer lithography. The device borrows from the principles of Purcell’s three-link swimmer,^50–54^ which is the simplest device capable of swimming in the Stokes regime.

Purcell’s three-link swimmer generates net motion in the Stokes regime by actuating three links connected by two hinges which are rotated out of phase with each other. This nonreciprocal motion is not symmetric in time and, thus, generates net fluid flow without relying on an inertial fluid. In our own Purcell-like system,^49^ the action of the hinges is replaced by the bending imposed upon three elasticated links being driven at the free end. The phase difference is generated passively by a lag between the links as the induced wave propagates down its length. The behavior of this class of elasticated swimmer can be reasonably described by the introduction of the sperm compliance number^55,56^ and this is referred to qualitatively throughout the analysis. The sperm compliance number is an important dimensionless metric relating the behavior of a damped elastic beam as a function of driving frequency, geometric parameters, and fluid resistance. Our pump is formed around an asymmetric rest position, avoiding fluid flow production parallel to the pumps length, and instead producing pronounced fluid flow in the perpendicular direction, allowing the pump to be tethered to a flat channel wall without complication.

The experimental device demonstrated flow rates appropriate for application; controllable and reversible with minor adjustments of the frequency of the driving field. Due to limitations of the experimental apparatus, only certain variables could be measured, namely, the motion path of the pump and the fluid velocity. Therefore, we were unable to fully characterize the system with measurements such as investigating the phase lag between driving torque and pump motion, a metric that has been implicated as an optimality criterion.^49,56^

Here, we use COMSOL Multiphysics,^57^ not only to verify experiment but also to test the dependence of device functionality on its surrounding channel walls and probe previously unattained parameters such as phase relationships. In this paper, we will present the results of these simulations and demonstrate the effects of modeling our experimental prototype to verify the previously observed behavior with our numerical model.

## METHODS

### Experimental synopsis

**FIG. 1.**
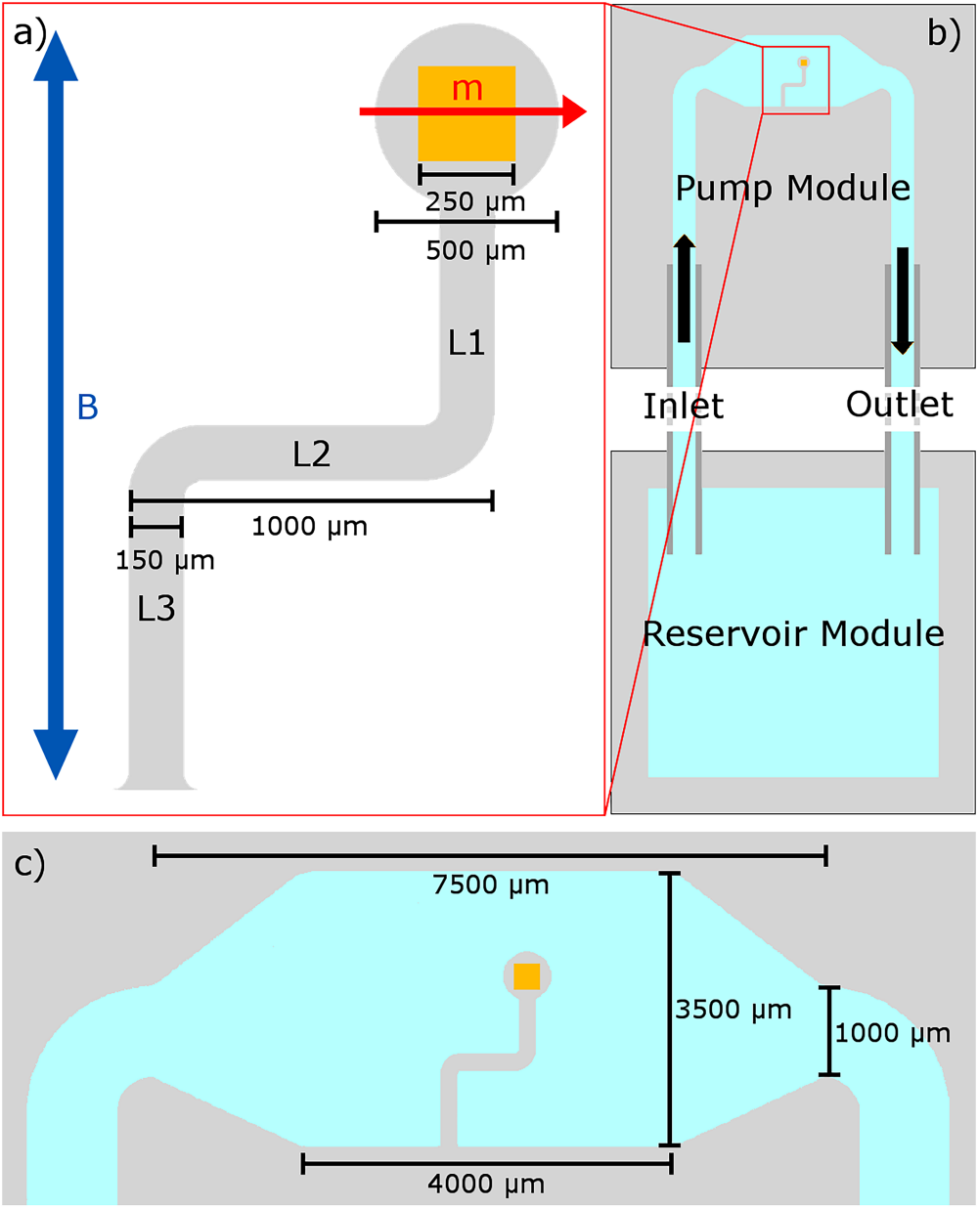
The geometry of the pump used in experiment. A diagram of the system explored in this study, depicting the pump, channels, and magnetic fields employed. The gray region signifies PDMS, the gold region signifies the NdFeB magnet of side length 250 μm, and the blue region signifies fluid. (a) Depicts the integrated pump. The pump consists of three elastic links, labeled L1 to L3. The width of the links is 150 μm, and the thickness is 300 μm. The blue arrow shows the direction of the uni-axial oscillating magnetic driving field, $\vec{B}$, and the red arrow shows the direction of magnetisation, $\vec{m}$, of the NdFeB magnet. (b) Depicts the device geometry. The device consists of a fully enclosed pumping module with the labeled inlet and outlet being square channels, 1000 μm in width and 900 μm in depth. The pump module is formed of three layers of PDMS, each with a feature size of 300 μm, the middle layer contains the integrated pump. The pump module is connected to an open reservoir module via PTFE tubing with a length of 4 cm and an internal diameter of 860 μm. The reservoir module allows for easy introduction of fluid and tracer particles during experiments. (c) Depicts the geometry of the pumping chamber with the dimensions displayed. [With permission from Binsley *et al.*, Lab on a Chip **20**, 4285–4295 (2020). Copyright 2020, Royal Society of Chemistry.]

These simulations are based on previous experiments from the same authors,^49^ and a brief summary is provided here: A microfluidic device with an embedded elasto-magnetic pump was fabricated from PDMS and an embedded NdFeB magnet (Fig. 1).

During experiments, the device was actuated using a uniform, uniaxial, sinusoidally oscillating external magnetic field supplied by a Helmholtz coil. The driving frequency and amplitude were varied, along with the fluid viscosity, in order to characterize the behavior of the device. The viscosity was controlled by altering a mixture of glycerol and water. 15 μm polystyrene beads were introduced to the device through the reservoir module in order to track the motion of the fluid and, therefore, calculate the fluid velocity and pumping effectiveness.

One of the key results found from the experiments was that under certain conditions the pump was able to produce fluid flow in the direction opposite to the pumping direction, although the exact cause for the effect was unknown. This is something hoped to be replicated and investigated through simulations.

### Numerical model

**FIG. 2.**
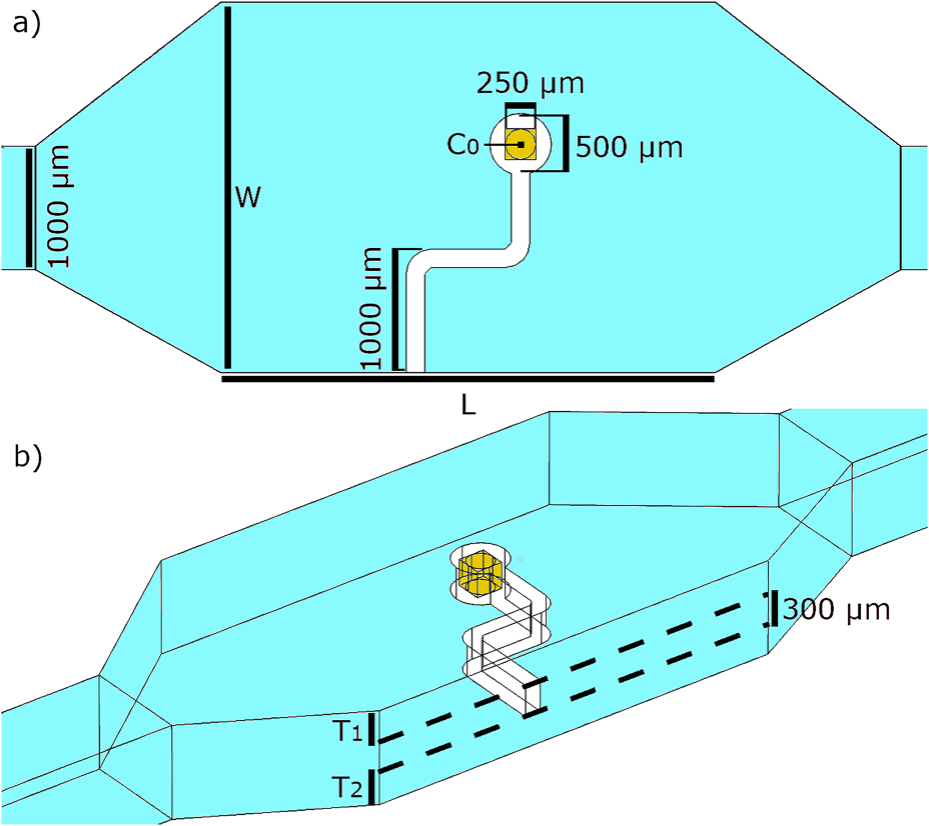
A schematic defining the geometry of the device. (a) Represents the in plane dimensions, L defines the length of the pumping chamber, and W defines the width of the pumping chamber, with other key distances defined as appropriate. (b) Represents the out of plane dimensions, showing the thickness of the pump and the thickness of the out of plane buffers on each side of the pump.

The geometry of the device (Fig. 2) was created to match the experimental system.^49^ This depicts the octagonal pumping chamber containing an elasto-magnetic pump, connected to a rectangular cross-section inlet and outlet of appropriate length, included to ensure appropriate hydraulic resistance. The octagonal shape of the pumping chamber is chosen to encourage impedance matching between the channels and the greater width required to accommodate the pump.

The design consists of three distinct planes with a standard thickness of 300 μm each: a middle plane containing the active pumping element, sandwiched between two buffer planes, setting the pump away from the channel walls and allowing motion in free space. The pump itself consists of three elasticated links, 1000 μm in length, joined at right angles to each other. Curved joinery allows for a constant link width, with an outer radius of 350 μm. A 250 μm square magnet is set inside the 500 μm diameter pump head. The pump is positioned within the pumping chamber such that the second (horizontal) link is centered on L, even when the length of this chamber is varied.

The existence of the out of plane buffer regions was important during experiments to allow a small amount of out of plane motion without the pump contacting the wall and stalling; this is particularly pertinent due to the large amplitude deformation of the unbounded end of the elastic armatures. This same buffer region is equally important here because as the pump deforms, so too must the mesh. The mesh is chosen to be stationary at the channel walls to ensure the integrity of the non-slip boundary condition and the stability of the model. Therefore, the thickness of the buffer regions determines the maximum distortion of the mesh elements and the resultant mesh quality. When these buffers are varied independently, the pump will move closer to one wall than the other, creating asymmetry along that axis.

The fluid is assumed to exhibit laminar flow with no-slip boundary conditions. The numerical simulations, therefore, solve for the incompressible Navier–Stokes equation and continuity equation with boundary conditions described in COMSOL as

$$\rho \frac{\partial u_{\;f}}{\partial t}+\rho (u_{\;f}\cdot \nabla )u_{\;f}=\nabla \cdot [-pI+K]+F,$$ (1)

$$\rho \nabla \cdot u_{\;f},$$ (2)

and

$$u_{\;f}(x=wall)=0,$$ (3)

where ρ is the density of the fluid, $u_{\;f}$ is the velocity of the fluid, p is the pressure, I is the identity matrix, and K is the viscous stress tensor. The simulations utilize linear discretization for the fluid velocity, linear discretization for the pressure, and 10^5^ tetrahedral mesh elements. This was chosen since increasing the discretisation and mesh elements did not significantly impact the solution, but did significantly increase computational expense. Remeshing was not included since it did not significantly affect the output trends, although it did increase the variance of the data points due to the introduction of discontinuities during interpolation of the solution between meshes.

The elasto-magnetic pump was modeled as a linear elastic material within COMSOL, including inertial terms, assuming Young’s modulus of 1.6 MPa,^58,59^ to match the experimental Young’s modulus. The magnetic driving field was not simulated within COMSOL but was instead an assumed total force acting tangential to the surface of a cylindrical insert within the magnet of the form

$$F=\Gamma S(t)\sin(\omega t)\cos(\theta )\;\hat{\theta },$$ (4)

where this force is related to the external magnetic driving field through

$$\frac{F}{r}=\;\tau =m\times B,$$ (5)

where Γ is the peak load, θ is the angle between the magnet and the driving field, t is the time, S is a smooth step function, r is the average effective radius of the magnet, τ is the torque about the magnet, m is the magnetic moment of the magnet, and B is the effective magnetic flux density of the driving field. This describes a sinusoidally oscillating magnetic field acting on a rotating magnet, with the force dependent on the angle between the magnet and the driving field. The step function has a step size equal to one period of the driving field, included to improve stability when beginning from a stationary zero point. Applying known analytical solutions such as Eq. (4) reduces model complexity while providing an idealized result.

### Solver details

The solver chosen was MUMPS, a direct solver. The Jacobian was set to update with every iteration to ensure a high accuracy solution in a dynamic system. The termination of the solver was dependent on iterations or tolerance, with a maximum of 15 iterations and a tolerance factor of 1.

The time stepping was strict and used the backwards differential formula (BDF)^60^ method with a maximum order of 2 and an event tolerance of 0.01. Consistent initialization was achieved using the backwards Euler method.

The geometry was meshed differently in two regions. The pump and pump chamber was meshed with tetrahedral elements, with sizes calibrated for fluid dynamics at “normal.” This resulted in a maximum mesh element size of 231 μm, a minimum mesh element size of 69 μm, a maximum element growth rate of 1.15, a curvature factor of 0.6, and a resolution of narrow regions set to 0.7. The extended straight channel was meshed similarly but calibrated to be “fine.” This resulted in a maximum mesh element size of 183 μm, a minimum element size of 34.5 μm, a maximum element growth rate of 1.13, a curvature factor of 0.5, and a resolution of narrow regions set to 0.8.

The discretization was kept at the default P1 + P1; linear in both velocity and pressure. Periodic boundary conditions are used to simulate a closed loop environment.

This mesh was chosen based on a mesh sweep, varying the mesh at various regions, and the discretization for each mesh. The aforementioned mesh and discretization was chosen since it showed only a 3.5% difference in normalized fluid displacement from the finest mesh tested, while still solving in a reasonable amount of time, and thus were the optimal parameters. The metric used to measure the behavior of the fluid is *normalized flow displacement*: the volume of fluid displaced per cycle divided by the volume swept by the pump. This is chosen instead of the simple, *averaged fluid velocity*, since the unitless result is agnostic to the compounding dependence on driving frequency, and provides a clearer picture of the impact of the pump motion.

### Numerical analysis

Simulations were continued for three oscillation periods including the step period. The final, third period was assumed to represent the steady state, with subsequent periods not significantly differing, and so only this period was used in the analysis.

**FIG. 3.**
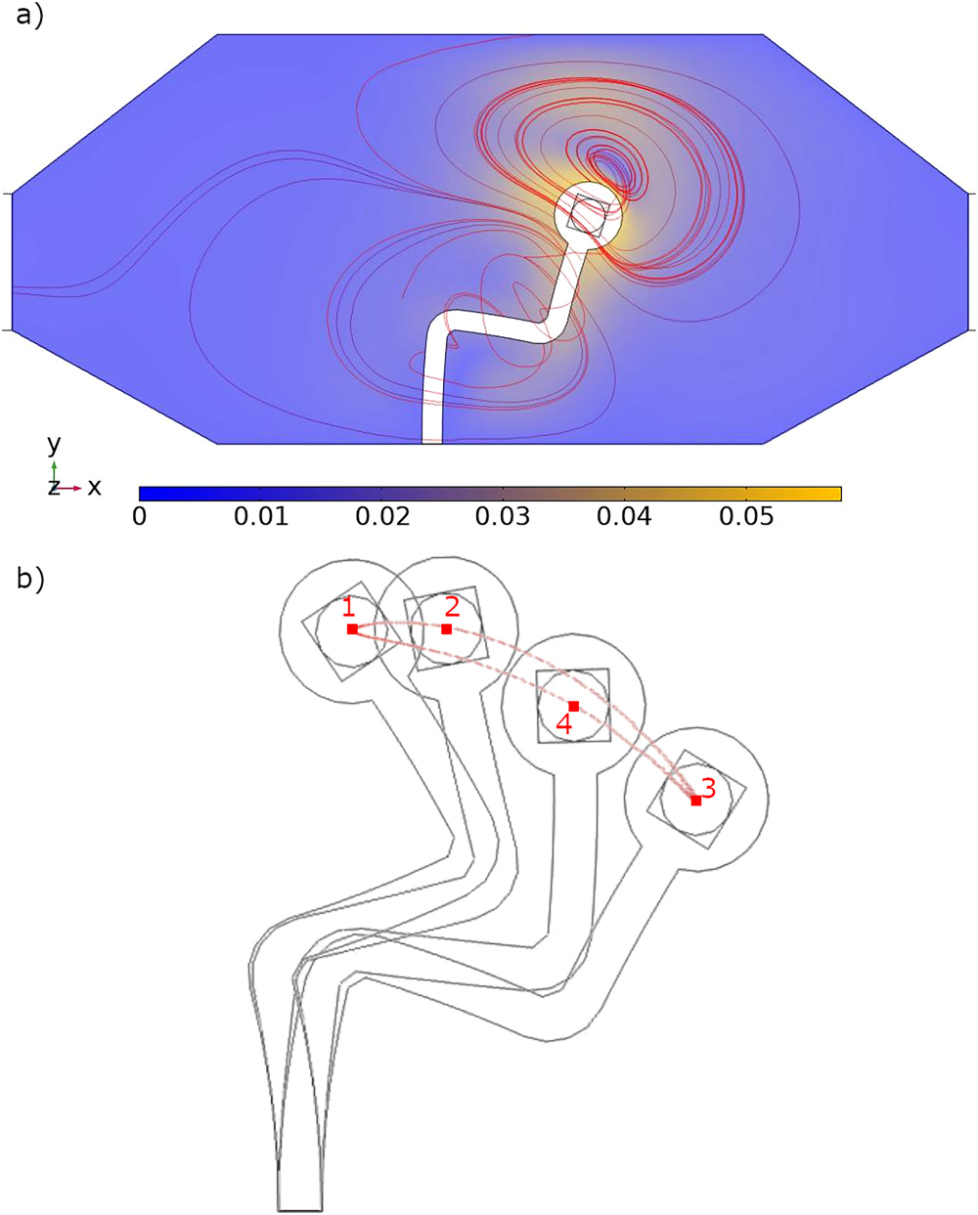
A figure depicting representative frames from the simulations. (a) shows the motion of the fluid, with a color map of the velocity magnitude of the fluid, with blue indicating low velocity and yellow depicting high velocity. Instantaneous streamlines are depicted in red as the fluid flows from the left to right. (b) Shows the motion of the pump over the course of one period of the cycle. The center of the pump head (the center of the magnet) follows the red closed loop path as it travels through 1 ⟶2 ⟶3 during the power stroke and through 3 ⟶4 ⟶1 during the recovery stroke.

The final cycle was separated equally into 300 time steps. At each time step, the fluid velocity at the inlet and outlet was recorded, along with the position of the center of the magnet, $C_{0}$ (Fig. 2). These 300 data points were further interpolated to accurately resolve key observed features. A maximum time step of 1/3000 s was used so as to respect CFL conditions. The motion of the $C_{0}$ was traced over time and its path plotted alongside representative streamlines showing the typical transient vortex structures created and the motion path responsible for it (Fig. 3).

## RESULTS

### The frequency of the driving field determines the net fluid flow

We start by verifying our model against experimental results from our previous study^49^ (Fig. 3). As previously, the pump is subjected to a uniaxial, sinusoidally oscillating magnetic field at a range of frequencies. We then measure the resultant motion of the pump by tracking the motion of the center of the pump head, $C_{0}$, through time, and the resultant flow rate produced at the channel inlet. The driving field amplitude was kept constant at an effective flux density of 6 mT. The viscosity and density of the fluid are also kept constant at 0.0077 Pa s and 1140 kg m^−3^, respectively. This is because the fluid is modeled as a mixture of glycerol and water, and the viscosity is controlled by adjusting the ratio of the two fluids. $T_{1,2}$, W, and L are 300, 3000, and 4000 μm, respectively.

The pump performs two distinct strokes, an extended power stroke and a retracted recovery stroke. The difference between these two strokes sweeps a closed loop path, enclosing a volume of fluid that must be displaced over the course of the action. As the pump head performs the power stroke, it follows a different path than when it is moving through the recovery stroke. In an open system, this would change the size and orientation of the vortices shown by the red streamlines (Fig. 3). The streamlines shown here are transient and the streamlines shown in the figure are only representative of a single point in time. Within this enclosed system, the vortices are restricted, and this path difference significantly alters the shape of the vortices between the power and recovery strokes. The presence of the power and recovery stroke emerges from the asymmetric rest position of the pump; if a straight, flexible design was used, the system would resemble Purcell’s flexible oar,^50^ and the path would resemble a symmetric curved “dumbell” shape. Such a stroke pattern would pump equally in both directions and create net flow only parallel to the length of the pump. With the asymmetric design, this instead mimics a two dimensional cilia.

**FIG. 4.**
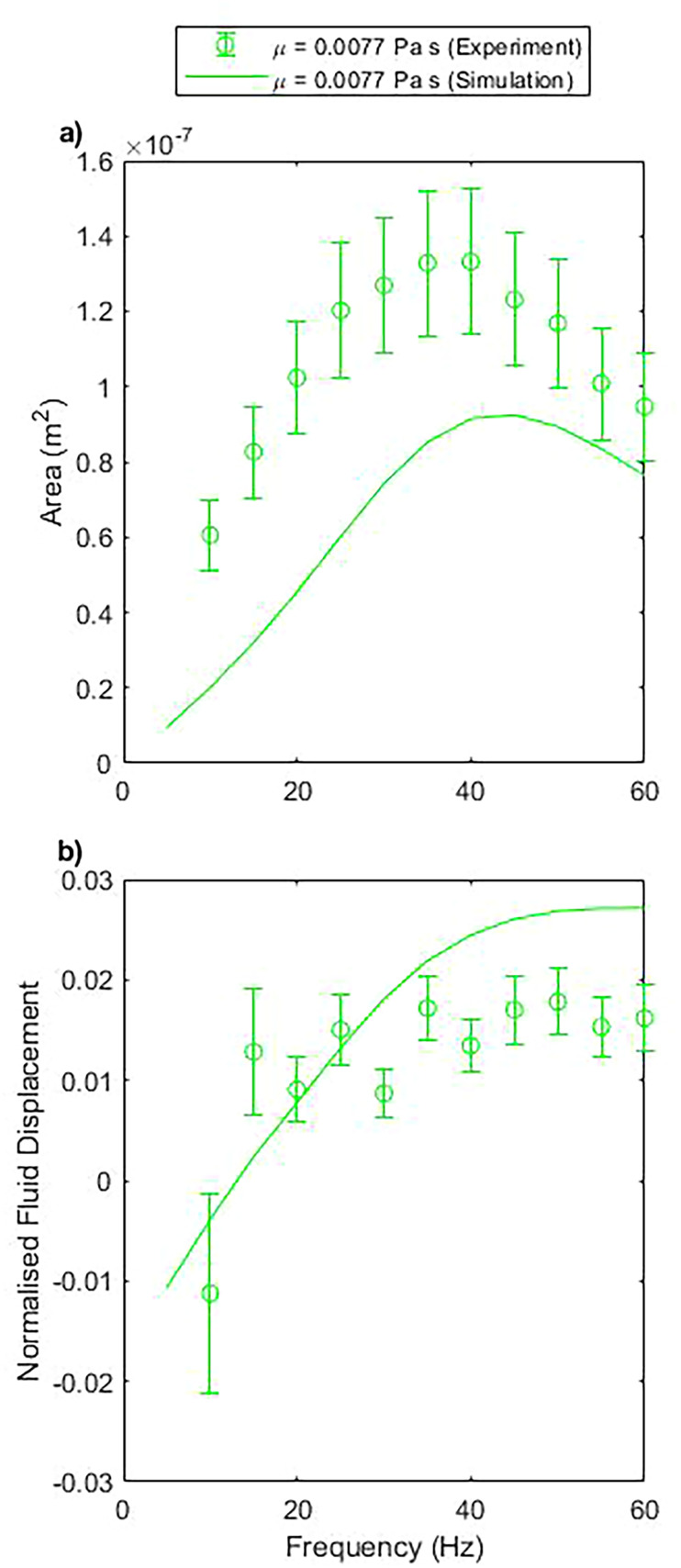
A comparison of simulation and experimental results. The error bar data are from experiment, while the line plots are from simulation data. Simulation and experiment show the same trends, with differing amplitudes of response. (a) Represents the area enclosed within the closed loop path performed by the pump head during one cycle as a function of frequency. (b) Represents the net fluid displacement per cycle divided by the volume swept by the pump.

The enclosed area swept by the pump motion increases from zero, at zero driving frequency, to a peak, and then begins to decrease at higher frequencies [Fig. 4(a)]. This is in accordance with an increasing sperm compliance number,^55,56^ with the pump motion being largely reciprocal at low driving frequencies; and an increasing driving frequency accentuates the non-reciprocity, as well as decreasing the total amplitude of motion. This quality is in agreement with the experimental results with only a discrepancy in the absolute magnitude of the response. The peak area measured experimentally is 44% larger than observed in simulation, and the peak area in simulation is also shifted to slightly higher frequencies.

When observing the normalized flow displacement [Fig. 4(b)], we find that both simulation data and experimental data asymptote toward a constant value at higher frequencies. At lower frequencies, the flow deviates from this value and both simulation and experiment show net flow in the negative direction at frequencies at and below 10 Hz, with a disagreement at 15 Hz, close to the zero point in simulation data. Again, the amplitude of the simulation data and experimental data does not exactly match, although the key observable features remain.

### Changing *W* changes the direction of flow

The presence of walls is implicated in perturbing the behavior of the fluid away from what would be expected from a three-link swimmer far from walls,^19,21^ although was not tested for this system experimentally. Therefore, a parameter sweep was conducted in simulation, altering the length ( L), width ( W), and thickness ( $T_{1}$ and $T_{2}$) of the pumping chamber, four parameters which are labeled in Fig. 3. L is varied from its original value of 4000 to 8000 μm; with fixed $T_{1,2}$ and W at 300 and 3000 μm respectively. W is varied from its original value of 3000–5000 μm; with fixed $T_{1,2}$ and L at 300 and 4000 μm, respectively. $T_{1}$ and $T_{2}$ are varied independently from a reduced value of 250–500 μm, with 300 μm being used in the experiment; with fixed L and W at 4000 and 3000 μm, respectively.

**FIG. 5.**
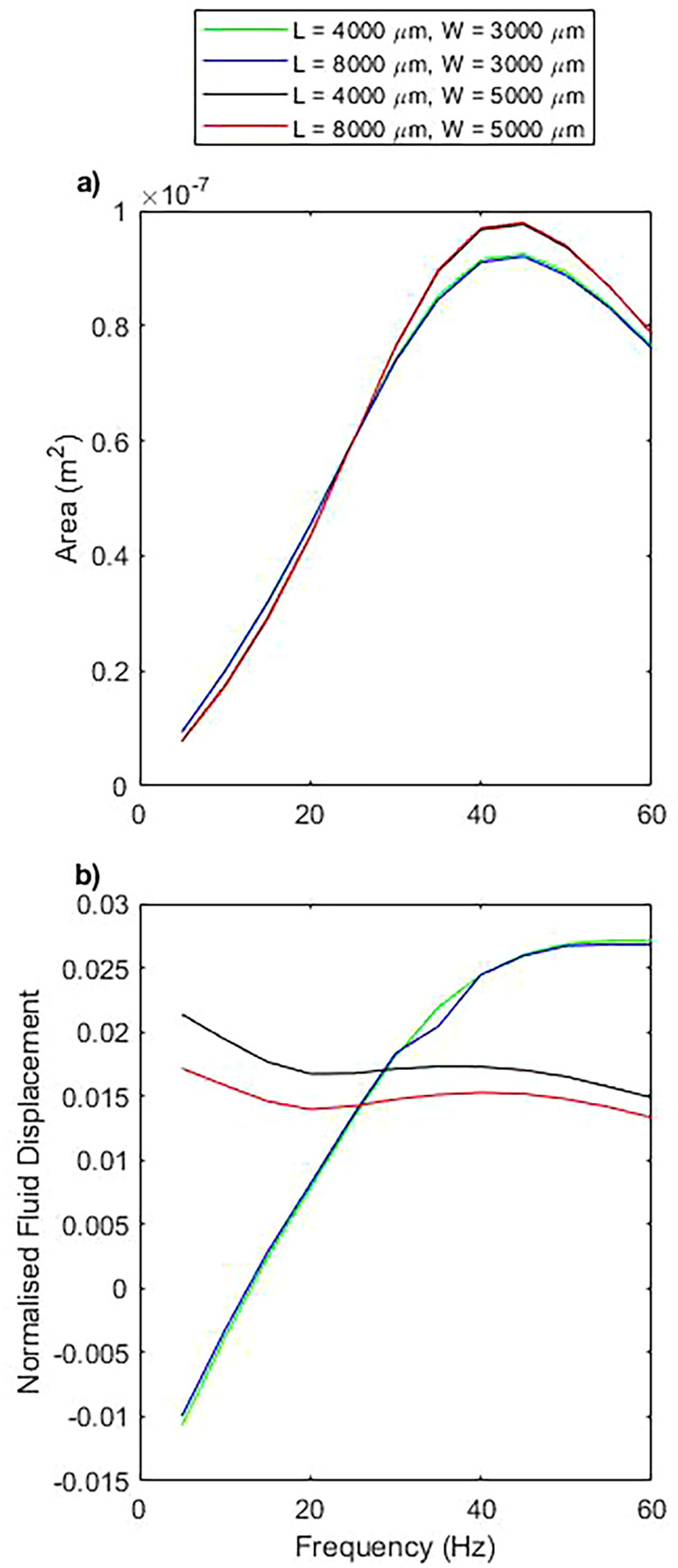
The impact of varying the in plane dimensions, L and W, as defined in Fig. 2. (a) Represents the area enclosed within the closed loop path performed by the pump head during one cycle for varying L and W, as a function of frequency. (b) Represents the volume of fluid displaced per cycle divided by the volume swept by the pump head as a function of frequency, for varying L and W, as a function of frequency.

When changing L between 4000 and 8000 μm, the area swept by the pump is unperturbed [Fig. 5(a)]. This indicates that the pump motion is not dependent on this distance as the length of the pump chamber behaves as a low resistance extension of the channel and is not strongly coupled to the pump. However, when increasing W from 3000 to 5000 μm, the area swept by the pump is increased for high frequencies and decreased for low frequencies, although the peak frequency is not altered. This change in area appears to be a result of decreasing the shear gradient across the fluid between the pump head and the relevant wall and, therefore, decreasing the resistance to motion.

The normalized flow displacement implies similar conclusions [Fig. 5(b)], with minimal dependence on L, since it only represents a low resistance extension of the flow channels, but a larger impact present when increasing W. Increasing W decreases the coupling to the bounding walls and the system tends toward the linearly proportional relationship between area swept and net flow production. This is indicative of the case of unbounded cilia-like pumps and the region of negative flow generation is, therefore, destroyed.^61^

**FIG. 6.**
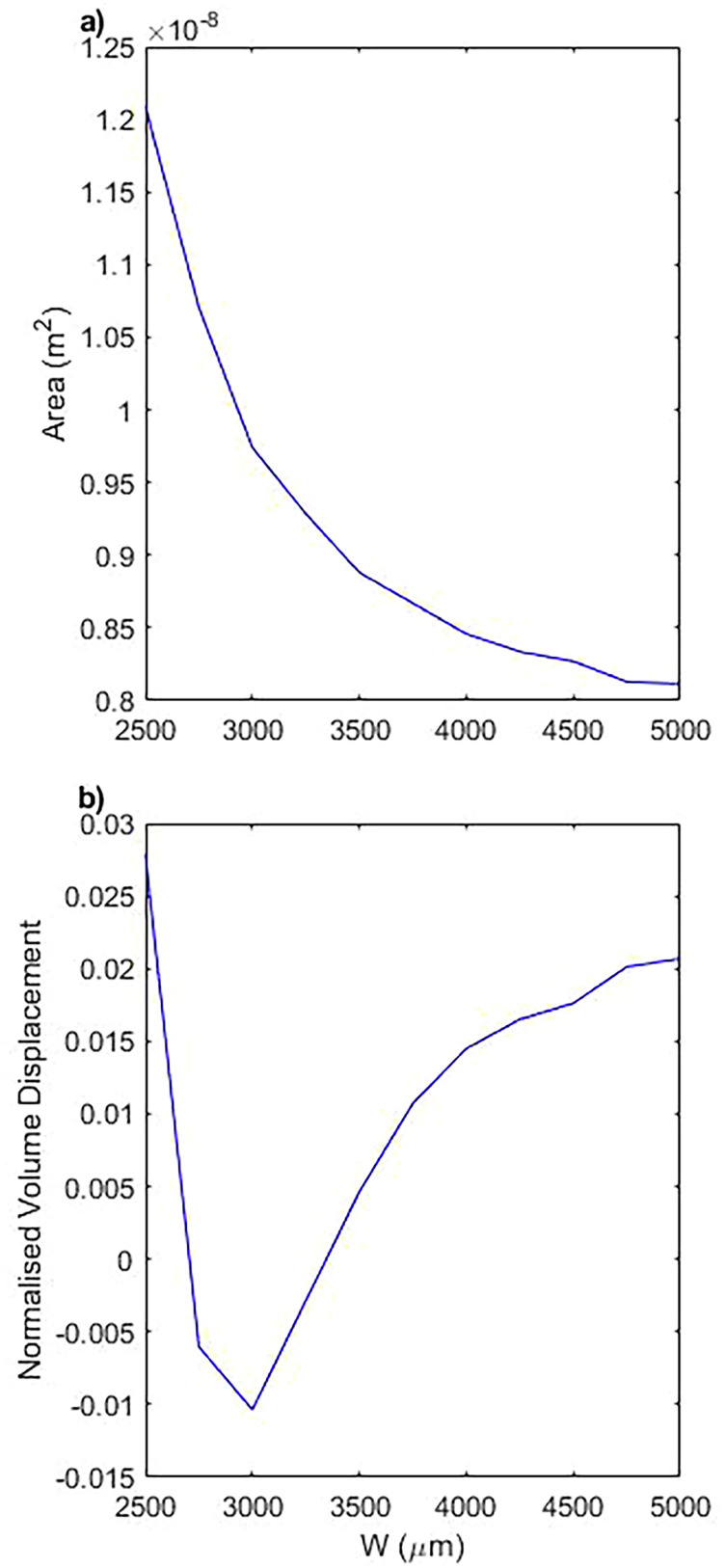
The impact of varying W at a fixed frequency. The chosen frequency is 5 Hz since it shows a region of interest. (a) Represents the area and (b) represents the normalized volume displacement.

The dependence on W is investigated further with a parametric sweep (Fig. 6), where we keep the driving frequency constant and change W between 2500 and 5000 μm. We choose frequency to be 5 Hz since this region shows the most distinct difference between the two previously shown curves (Fig. 5). The enclosed area shows a distinct change, decreasing with increasing W: the shape of the closed loop path traced by $C_{0}$ becomes narrower in the direction of W and longer in the direction of L. This change is seen since the shear rate is reduced, reducing the resistance to motion attributed by the fluid.

The impact that this has on the fluid is seemingly non-linear, with a peak deviation in normalized volume displacement occurring at 3000 μm (Fig. 6). Increasing W above 3000 μm the trend increases logarithmically and decreasing W below 2750 μm returns sharply toward the positive net fluid flow. The exact reason for this low W behavior is still undetermined, although since the tip of the pump reaches 2087 μm into the channel at rest, the minimum W of 2500 μm becomes very close to the tip of the pump in motion.

**FIG. 7.**
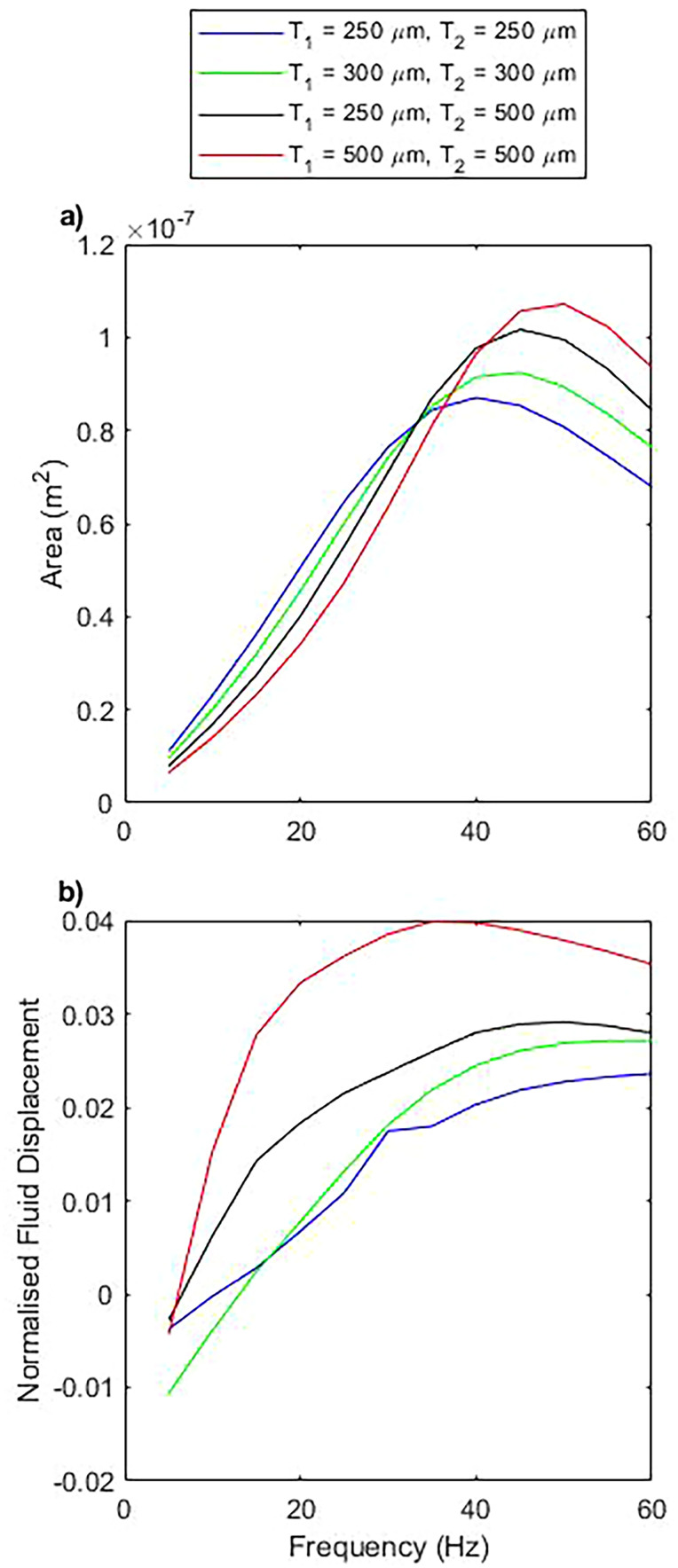
The impact of varying the thickness of the two out of plane buffers $T_{1,2}$, as defined in Fig. 2. (a) Represents the area enclosed within the closed loop path performed by the pump head during one cycle for varying $T_{1}$ and $T_{2}$, as a function of frequency. (b) Represents the normalized fluid displacement for varying $T_{1}$ and $T_{2}$.

It is also found that the motion of the pump is strongly dependent on $T_{1}$ and $T_{2}$, the out of plane thickness either side of the pump that acts as “head room” [Fig. 7(a)]. Increasing the out of plane dimensions of the pumping chamber increases the area swept by $C_{0}$, which is again attributed to a reduction in the shear rate and, therefore, a reduction in the resistance to motion. In this case, we also see a shift in the peak area toward higher frequencies, which is indicative of a reduced sperm compliance number as a result of the lower resistance. The hydraulic resistance of the chamber varies between $3.7\times 10^{7}$ Pa s m ${}^{-3}$ at minimum $T_{1,2}$ and $1.0\times 10^{7}$ Pa s m ${}^{-3}$ at maximum $T_{1,2}$. The distance between the out of plane walls and the moving pump is consistently the shortest distance between the pump and any surface and, therefore, it is reasonable that the pump will be most sensitive to changes in this dimension. We see the opposite effect when decreasing the out of plane dimension, with a reduced peak area, shifted toward lower frequencies. Altering $T_{1}$ and $T_{2}$ independently appears to offer no new behavior for the case tested here and simply acts as the symmetric case with $T_{1}$ = $T_{2}$ as an intermediate value between the two.

This is mimicked in the normalized fluid displacement; the increased out of plane thickness reduces the resistance to flow and so increases the flow rate. Since the hydraulic resistance is dominated by the channel extensions either side of the pumping chamber, we can see a strong correlation between the normalized fluid displacement and the hydraulic resistance of the channels with varying thickness, with the total hydraulic resistance varying between $1.9\times 10^{9}$ Pa s m ${}^{-3}$ at minimum $T_{1,2}$ and $1.2\times 10^{9}$ Pa s m ${}^{-3}$ at maximum $T_{1,2}$. The shape of this curve is preserved since there is minimal out of plane fluid flow, even at maximum $T_{1,2}$. This is supported when considering an asymmetric channel thickness, which does not change the behavior of the system and behaves only as the equivalent symmetric system.

Increasing $T_{1}$ and $T_{2}$ corresponds to a shift in the normalized flow displacement, but even when increased to the maximum, the relationship between area and flow rate remains non-linear [Fig. 7(b)]. This is because, since there is minimal flow produced in this out of plane direction, the change does not significantly perturb the streamlines, only the resistance to flow.

### Changing the hydraulic resistance does not change the direction of flow

**FIG. 8.**
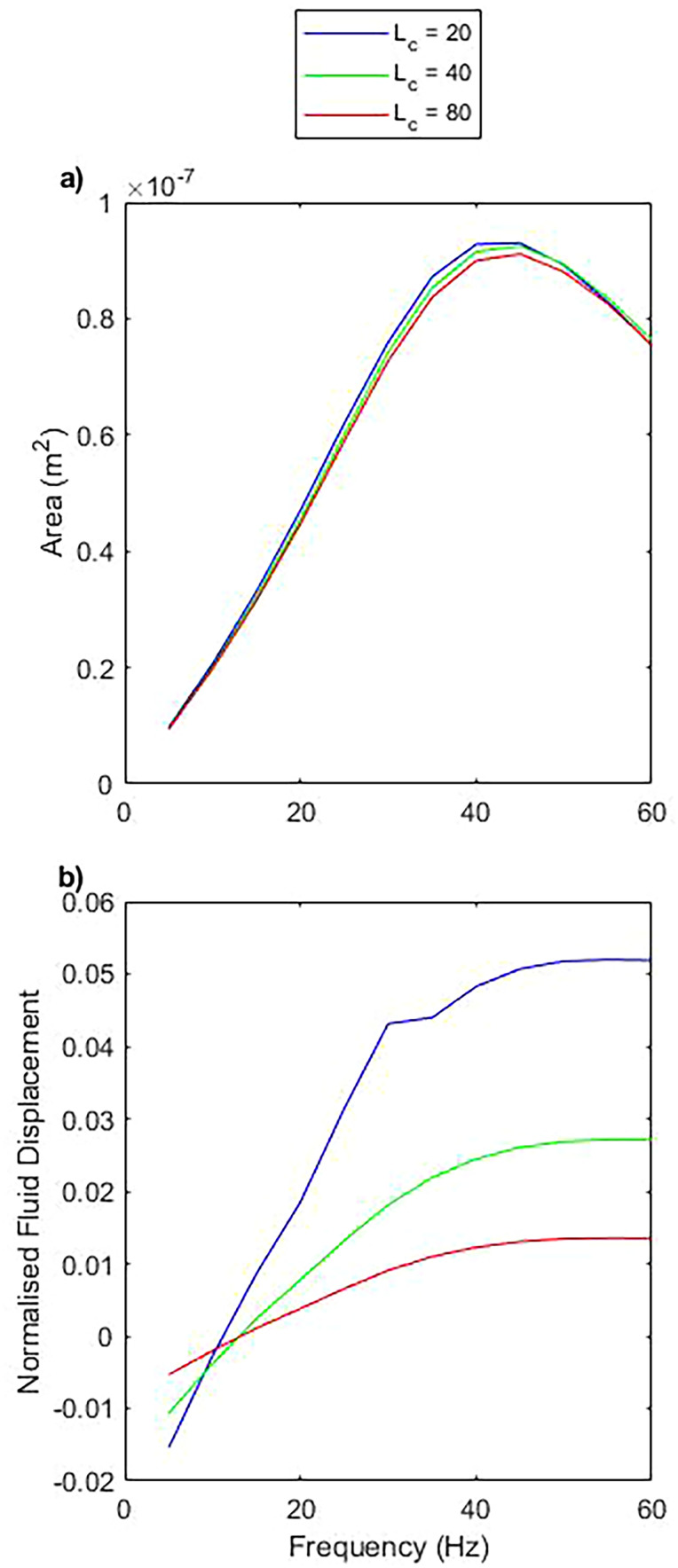
The impact of varying the length of the channels protruding from the pumping chamber. (a) Represents the area enclosed within the closed loop path performed by the pump head during one cycle, for varying channel length, as a function of frequency. (b) Represents the normalized fluid displacement for varying channel length.

To explore how other components of a microfluidic device may impact the behavior and performance of the pump, we varied the length of the extended channels, varying the resultant hydraulic resistance of the system (Fig. 8). The geometry of the pumping chamber is kept constant with $T_{1,2}$, W, and L at 300, 3000, and 4000 μm, respectively.

The initial channel length is 40 mm each side, totaling 80 mm of channel, similar to that of the experiment. When the total channel length is increased to 160 mm or reduced to 40 mm, we see minimal difference in the motion of the pump, while the normalized fluid displacement only changes in amplitude, not trend. This is reasonable when considering that driving a fluid through a channel with greater hydraulic resistance, with the same pressure, should result in a decreased flow rate. As the contribution to the hydraulic resistance is dominated by the length of these channels (by ∼2 orders of magnitude), doubling the channel length corresponds to doubling the hydraulic resistance, and can be seen here to approximately halve the fluid flow. When $L_{c}=40$, the hydraulic resistance can be calculated as $1.5\times 10^{9}$.

### Phase difference between the driving field, pump motion, and fluid flow

**FIG. 9.**
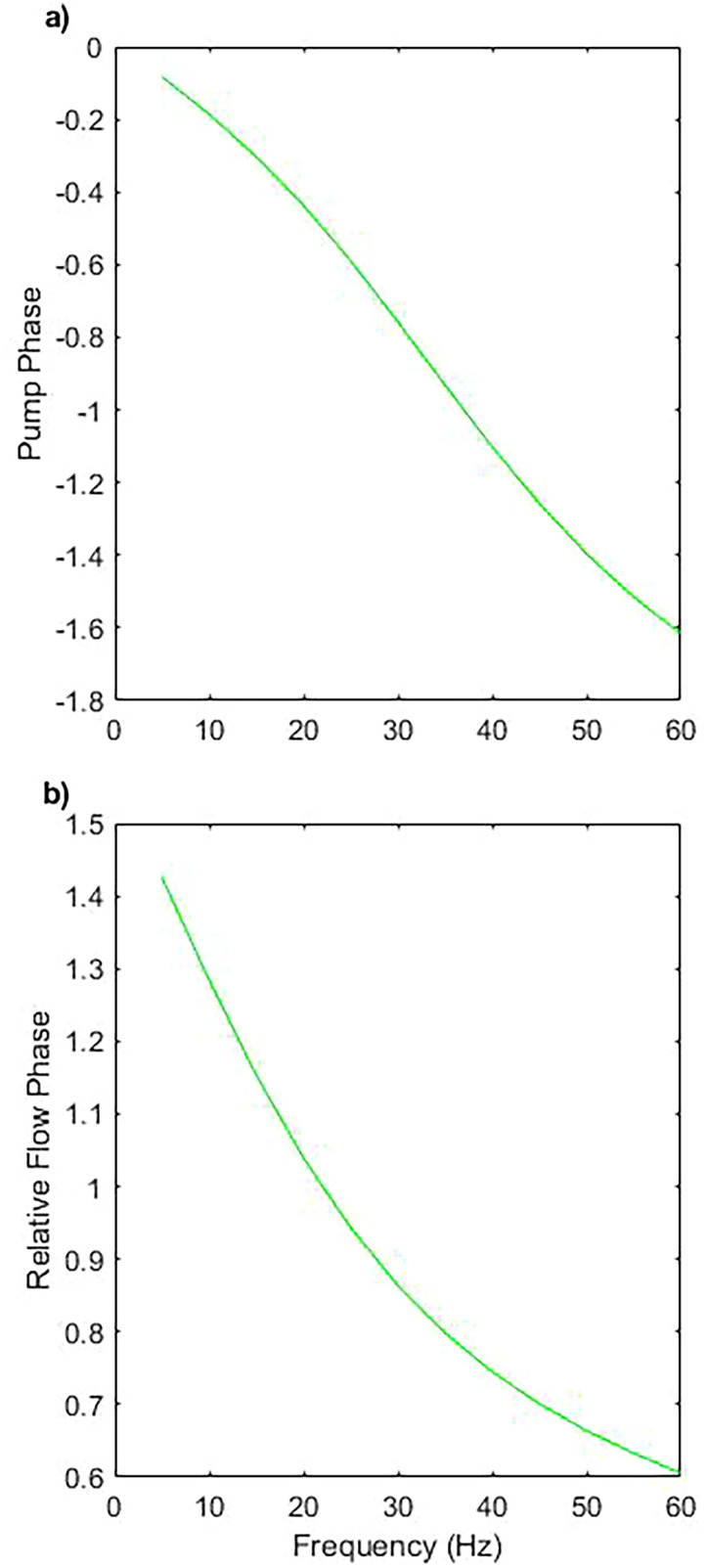
(a) Phase difference between the motion of the pump head and the driving field as a function of frequency for varying fluid viscosities. (b) Phase difference between fluid flow and motion of the pump head for varying fluid viscosities.

The phase differences between the driving field, the pump motion, and the fluid flow are important key metrics in understanding the behavior of this system. Since the motion of the elastic component is not quasistatic, described by the finite Reynolds number, its motion must lag behind the driving field. The expectation is that as the frequency increases, so too does the phase lag between the driving field and the motion of the pump. The phase difference between the driving field and the pump motion are measured for the case of $T_{1,2}$, W, and L as 300, 3000, and 4000 μm, respectively, along with the phase difference between the pump motion and the resultant fluid flow, as a function of frequency [Fig. 9(a)].

The phase difference between the driving field and the pump motion is calculated as a function of frequency and we observe that at low frequencies, the phase difference tends to zero, with the pump head returning to the zero position when no driving force is applied. However, as the frequency increases, the pump naturally begins to lag behind the driving field due to the possession of a finite response time, with the finite acceleration of the pump responsible for breaking time symmetry.

A different impact is found when considering the phase difference between the pump motion and the fluid flow [Fig. 9(b)]. At low frequencies, the flow is generated π/2 ahead of the pump motion since the power stroke begins at maximum displacement rather than the rest position of the pump. A phase lag emerges as driving frequency increases despite the low Reynolds number.

A phase lag is expected for a system with a finite Womersley number, α,^62,63^ which implies that the flow is oscillating rapidly enough that the typical parabolic flow profile is not able to fully form before it is destroyed and reversed. This narrows the boundary layers and causes the flow rate to lag in phase behind the driving pressure. For flow in a rigid, straight channel, a maximum phase lag of π/2 arises at α→∞. It has, however, been shown that altering the geometry can increase the phase lag beyond π/4 or reduce it below 0.^63^ The emergence of this change in phase lag is attributed to orthogonal fluid flows.

The Womersley numbers calculated at the inlet/outlet are, however, less than 1 for all cases, and we can assume that the flow profile is adequately developed over the course of each cycle far from the pump. For flow inside the pumping chamber, simple analytic expressions would offer an inaccurate description. The emergence of this significant phase lag is attributed to the topological impacts of fluid flow near to the pump and further investigations are beyond the scope of this paper.

## DISCUSSION

A clear correlation is found between the width of the pumping chamber and the net fluid flow, with small changes in W altering the pumping direction (Fig. 6). The pumping direction is, however, robust for changes in L and T. From this, we can prove that the shape of the chamber is just as important as an optimization criterion as the design of the pump itself, with even small variations having a large impact on overall performance. This confirms our original hypothesis that the phenomena observed arise from the near vicinity of the bounding walls, but also suggest the requirement of fine engineering tolerances when attempting to accurately reproduce the system. The flow produced by the pump lags behind the motion of the pump, moreso than the Womersley number would suggest. The emergence of this phase is, therefore, an area of interest for further investigation and phase data should be considered an important optimization criterion.

These simulations are satisfactorily verified against experiment (Fig. 4). The absolute area swept is of the same order of magnitude, with an understandable discrepancy when considering the approximations and fabrication tolerances involved. The ability to produce flow in the negative direction is preserved for the base case and confirms that its existence is not anomalous. This negative region occurring at low frequencies does, however, mean that the resultant negative flow rate is very low.

These simulations are relevant beyond this specific application as they help to describe a key emergent phenomenon of moving elastic systems such as cilia in a confined domain which is not often considered, and further work should be encouraged to investigate if biologically accurate cilia exhibit the same trends under confined situations: since this phase relationship may have considerable effects on optimizing emergent metachronal activity.^47,48^

The comparisons made between this system and alternative experimental systems made previously^49^ are largely preserved. The device is designed to operate within the fully enclosed environment of microfluidic channels, which is not proven for all elasto-magnetic pumping designs.^18,21^ A simple and commonly produced remote driving field is all that is required to control the behavior of the system, producing a range of flow rates, oscillation ratios, and net flow directions; not relying on complex control systems that introduce barriers to their actuation outside of laboratory environments. No external connections are required and so fully enclosed, circular systems can be produced and maintained, without imposing any chemical changes upon the system.

The ability to create significant pulsatile flow opens avenues for the usage of the device in organ-on-a-chip and micromixing applications;^64,65^ although this is less appropriate where static pressures are desired, such as cell trapping.^12^ This, therefore, indicates a good candidate for integrated pumping within microfluidic LOC devices for POC applications.

## CONCLUSIONS

This work successfully models an elasto-magnetic Purcell-like integrated pumping system for microfluidic applications. This model is verified against experimental results and garners confidence in the reproducibility of this design as a mechanism for pumping. A clear interaction between the fluid flow and the surrounding channel walls is shown, where a more restricted system, increasing this interaction, is capable of encouraging flow in the reverse direction. The interaction highlights the channel walls as an important point of consideration when optimizing this design.

Access to parameters such as the phase difference between pump motion and fluid flow has enabled insight into the behavior of this system, opening the door for future investigations. This will be important for potential future work such as introducing an array of these devices and investigating the emergence of metachronal waves.

## Data Availability

The research data supporting this publication are openly available from the University of Exeter's institutional repository at https://doi.org/10.24378/exe4264, Ref. 66.
